# RhoGEF Tiam2 Regulates Glutamatergic Synaptic Transmission in Hippocampal CA1 Pyramidal Neurons

**DOI:** 10.1523/ENEURO.0500-21.2024

**Published:** 2024-07-16

**Authors:** Sadhna Rao, Feng Liang, Bruce E. Herring

**Affiliations:** Department of Biological Sciences, Neurobiology Section, Dornsife College of Letters, Arts and Sciences, University of Southern California, Los Angeles, California 90089

**Keywords:** development, glutamatergic, hippocampus, regulation, spines, synapse

## Abstract

Glutamatergic synapses exhibit significant molecular diversity, but circuit-specific mechanisms that underlie synaptic regulation are not well characterized. Prior reports show that Rho-guanine nucleotide exchange factor (RhoGEF) Tiam1 regulates perforant path→dentate gyrus granule neuron synapses. In the present study, we report Tiam1's homolog Tiam2 is implicated in glutamatergic neurotransmission in CA1 pyramidal neurons. We find that Tiam2 regulates evoked excitatory glutamatergic currents via a postsynaptic mechanism mediated by the catalytic Dbl-homology domain. Overall, we present evidence for RhoGEF Tiam2's role in glutamatergic synapse function at Schaffer collateral→CA1 pyramidal neuron synapses.

## Significance Statement

Glutamatergic synapses are known to vary in composition and function, but how this heterogeneity is established to create input-specific synaptic diversity is not well understood. In the present study, we show Tiam2 regulates glutamatergic neurotransmission at Schaffer collateral→CA1 pyramidal neuron synapses. We find that this function is dependent on its catalytic domain. By contrast we did not observe a role for Tiam2 in synaptic transmission at perforant path→DG granule neuron synapses. We also find that Tiam1 and Tiam2 are individually dispensable for functional synaptic plasticity in CA1 pyramidal neurons. To our knowledge, this is the first evidence of the Rho-guanine nucleotide exchange factor Tiam2's role in regulating glutamatergic synapses.

## Introduction

Recent studies have highlighted the molecular heterogeneity of glutamatergic synapses (Wichmann and Kuner, 2022). Transcriptomic and proteomic profiling has revealed cell type- and input-specific diversity in synaptic molecular composition (Zhao et al., 2001; Fort and Blangy, 2017; Zhu et al., 2018; Farris et al., 2019; Gerber et al., 2019; Franjic et al., 2022). However, an understanding of how synapse maturation, circuit assembly, and plasticity are regulated by distinct synaptic proteomes presents a significant challenge (Gutman-Wei and Brown, 2021; Marcassa et al., 2023).

Rho-guanine nucleotide exchange factors (RhoGEFs) are widely implicated in molecular mechanisms essential to the assembly and function of glutamatergic synapses (Kang and Schuman, 1995; Penzes et al., 2001, 2003; Fu et al., 2007; Margolis et al., 2010; Herring and Nicoll, 2016; Hamilton et al., 2017; Martin-Vilchez et al., 2017). RhoGEFs contain a catalytic Dbl-homology (DH) domain that catalyzes small-GTPase activity and promotes actin polymerization within postsynaptic compartments (Karnoub et al., 2001; Miller et al., 2013). Previous work has shown that the RhoGEF Tiam1 is important for the function of glutamatergic synapses at perforant path→dentate gyrus (DG) granule neuron synapses (Rao et al., 2019; Cheng et al., 2021). In the present study, we describe a functional role of the homologous protein Tiam2 at Schaffer collateral–CA1 pyramidal neurons.

Previous reports have characterized Tiam2's role in cell migration and neurite extension in vitro (Matsuo et al., 2002; Yoshizawa et al., 2002; Chan et al., 2020). We find that shRNA-mediated depletion of Tiam2 in CA1 pyramidal neurons results in significant reductions in AMPA receptor (AMPAR)- and NMDA receptor (NMDAR)-mediated evoked currents. We establish that postsynaptic Tiam2 expression contributes to glutamatergic neurotransmission and that Tiam2's effect on neurotransmission at Schaffer collateral→CA1 pyramidal neuron synapses depends on its catalytic DH1 domain. Tiam1 shares significant DH1 domain homology with Tiam2, and we show that recombinant Tiam1 expression can readily substitute for Tiam2 in supporting glutamatergic neurotransmission at this synapse. Additionally, our data also suggest no role for Tiam2 in neurotransmission at perforant path→DG granule neuron synapses. Moreover, we find that Tiam1 and Tiam2 are individually dispensable for functional long-term potentiation (LTP) at Schaffer collateral→CA1 pyramidal neuron synapses. In summary, we find that Tiam2 regulates baseline glutamatergic neurotransmission at Schaffer collateral→CA1 pyramidal neuron synapses.

## Materials and Methods

### Experimental constructs

**Figure 1.**
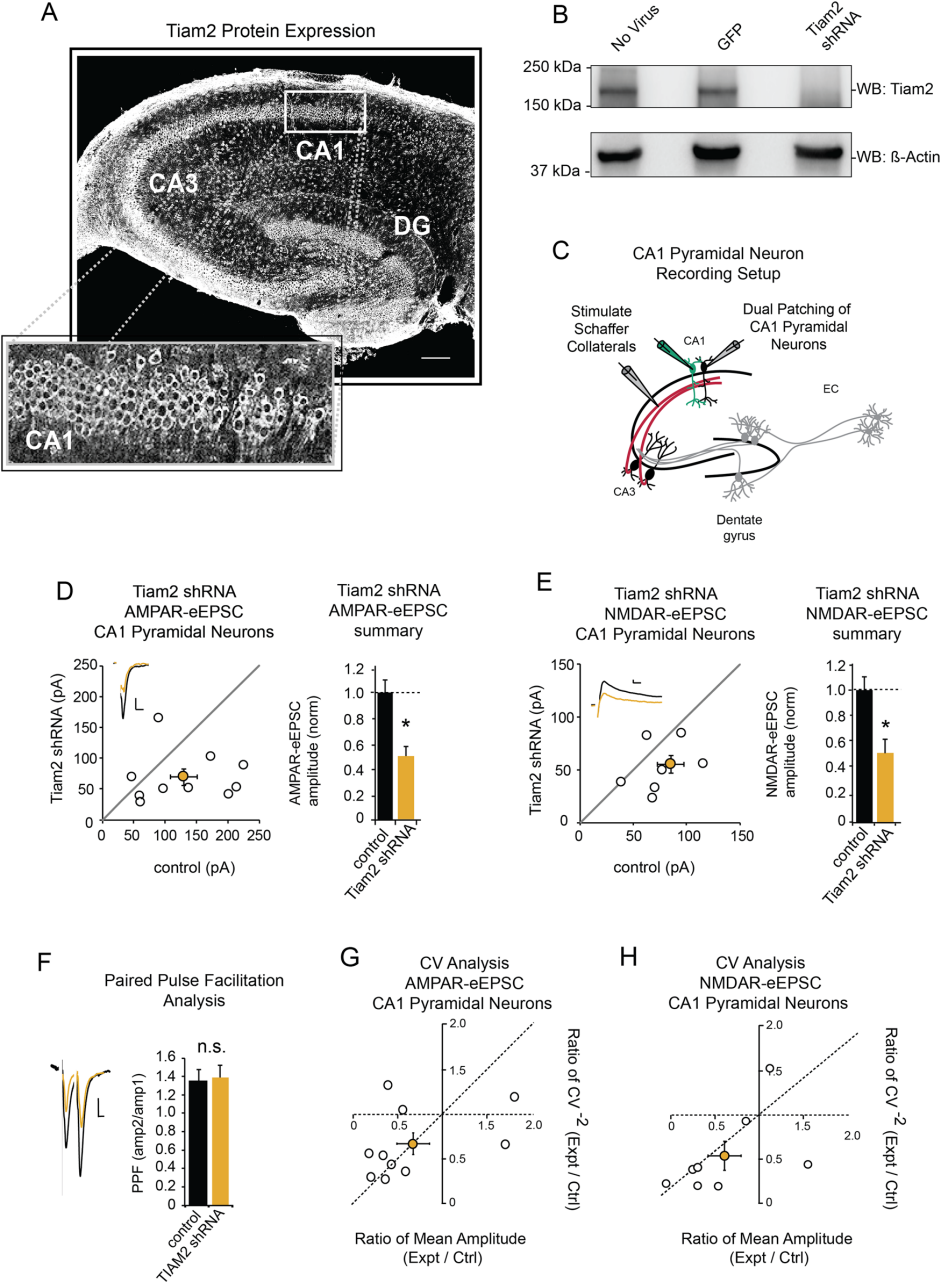
Tiam2 knockdown reduces baseline AMPAR- and NMDAR-mediated neurotransmission in CA1 pyramidal neurons. ***A***, Tiam2 immunolabeling in whole hippocampal slice, (inset) CA1 cell body layer (scale bar, 200 μm). ***B***, Western blot showing shRNA-mediated reduction of Tiam2 expression in dissociated rat hippocampal neurons. ***C***, Schematic representation of electrophysiological recording setup for CA1 pyramidal neurons. ***D***, ***E***, Scatterplots show eEPSC amplitudes for pairs of untransfected and transfected cells (open circles) with *M* ± SEM (filled circles). (Insets) Representative current traces from control (black) and transfected (yellow) neurons with stimulation artifacts removed (scale bars: 20 pA for both AMPAR-eEPSCs and NMDAR-eEPSCs, 20 ms for AMPAR, 50 ms for NMDAR). Barplots show average AMPAR and NMDAR-eEPSC amplitudes (±SEM) of CA1 pyramidal neurons expressing Tiam2 shRNA (yellow) normalized to their respective control cell average eEPSC amplitudes (black). Tiam2 shRNA expression decreases AMPAR-eEPSC and NMDAR-eEPSC amplitude in CA1 pyramidal neurons (*p* = 0.048; *n* = 10 pairs for AMPAR-eEPSC; *p* = 0.038; *n* = 8 pairs for NMDAR-eEPSC; Wilcoxon signed-rank test). ***F***, Paired-pulse facilitation ratio (*M* ± SEM) for Tiam2 shRNA expressing CA1 pyramidal neurons and paired control neurons with no detectable difference in facilitation (*p* = 0.88; *n* = 5 pairs; Student's unpaired *t* test). Representative scaled current traces from control and transfected (yellow) neurons (scale bars: 20 pA, 20 ms). ***G***, ***H***, CV analysis of AMPAR- and NMDAR-eEPSCs from pairs of control and Tiam2 shRNA expressing CA1 pyramidal neurons. CV^−2^ values are plotted against corresponding ratios of *M* amplitudes within each pair (open circles) with *M* ± SEM (filled circle; for AMPAR-eEPSCs, *n* = 10 pairs; for NMDAR-eEPSC, *n* = 8 pairs,). *p < 0.05, n.s., not significant. See Extended Data Figure 1-1 for more details.

A previously characterized Tiam2 shRNA target sequence against rat Tiam2 was used (5′-GGAGCTGCCTTTCTCACTTTA-3; Goto et al., 2011). The Tiam2 shRNA was subcloned behind the H1 promoter region of a GFP-expressing pFHUGW expression vector. A single-nucleotide mismatch was identified in the corresponding mouse Tiam2 target sequence. However, Western blot analysis confirmed effective and specific knockdown of Tiam2 expression by the rat-directed shRNA in both rat and mouse neurons (Fig. 1*B* and Extended Data Fig. 4-1*B*,*C*).

10.1523/ENEURO.0500-21.2024.f1-1 Figure 1-1 **Antibody and shRNA validation A** Tiam1 immunolabelling (white) in whole hippocampal slice co-localized with DAPI (blue), peptide control shows specificity of Tiam2 antibody (right panel) (scale bar: 500μm). **B** Immunoblot of DIV 21 rat hippocampal lysate transduced with no virus, GFP- and Tiam2 shRNA- expressing virus probed with Tiam2 antibody and actin (control). Download Figure 1-1, TIF file.

All cloning was performed using overlap-extension PCR followed by In-Fusion Cloning (Clontech Laboratories). The shRNA-resistant Tiam2 was generated by introducing five silent-point mutations within the RNAi target sequence in Tiam2 cDNA (GAAGTTGTCTATCACACTTTA). Tiam1 cDNA was from Horizon Discovery (catalog #MHS6278-211691099), and Tiam2 cDNA was from GenScript Biotech (catalog #ORa14059). cDNA sequences were cloned into pCAGGS-IRES-mCherry expression vectors. The Tiam2 ΔDH mutant was generated by deletion of the ∼200-residue DH1 domain (Arg1117-Met1312) using overlap-extension PCR followed by In-Fusion Cloning (Clontech Laboratories) into a pCAGGS-IRES-mCherry expression vector. Expression constructs were coexpressed with a pFHUG vector containing GFP, which also served as a control vector for spine imaging experiments.

### Tissue harvest and immunohistochemistry

All animal procedures were performed in accordance with the University of South California animal care committee's regulations. Postnatal Day (P) 15 rats were perfused with 4% paraformaldehyde via transcardial perfusion following which hippocampi were dissected and fixed in 4% PFA overnight at 4°C and then transferred into 1XPBS solution. Fixed tissue was sliced to 100–150 µm slices using a MX-TS tissue slicer (Siskiyou). Tissue sections were probed with antibodies (mouse monoclonal anti-Tiam2, 1:100, sc-514090, Santa Cruz Biotechnology; sheep anti-Tiam1, 1:100 AF5038, R&D Systems; and anti-Prox1, 1:2000, ab5475, Merck Millipore). For peptide control experiments, antibody was incubated with a blocking peptide (sc-514090 P) at 1:1 ratio overnight at 4°C and applied to samples in parallel with antibody staining. Fluorophore-coupled secondary antibodies were used for antibody detection, and slides were imaged using a Zeiss LSM-780 inverted microscope equipped with a 10×/0.45 M27 objective. Tiled 16-bit images were processed using Zen (Zeiss) to produce a single image file.

### Organotypic slice preparation and electrophysiology

P6 to P8 Sprague Dawley rats of both sexes were used to prepare organotypic hippocampal slice cultures as previously described (Stoppini et al., 1991; Prang et al., 2001; Bonnici and Kapfhammer, 2009). Hippocampal tissue with and without attached entorhinal cortical tissue were harvested from six to eight rat pups, and an MX-TS tissue slicer (Siskiyou) was used to prepare 400 μm transverse sections. Tissue slices were placed on squares of Biopore Membrane Filter Roll (Merck Millipore, catalog #BGCM00010) and placed on Millicell Cell Culture inserts (Merck Millipore) in 35 mm dishes containing 1 ml culture media (MEM + HEPES, Invitrogen 12360-038, 25% horse serum, 25% HBSS, and 1 mM L-glutamine). Media were replaced on alternate days.

Biolistic transfections were performed on DIV1 as previously described (Stoppini et al., 1991; Schnell et al., 2002; W. Lu et al., 2009). Briefly, 1-µm-diameter gold particles were coated with 50 µg plasmid DNA via coprecipitation with CaCl_2_, serially washed with ethanol. DNA-coated gold particles were coated onto the inner surface of PVC tubing, dried using nitrogen gas, and used for DNA delivery with a Helios Gene Gun (Bio-Rad Laboratories). Recordings were made on DIV 7 or 9 in organotypic slice cultures at room temperature (RT). The recording chamber was perfused at 2.5 ml/min^−1^ with artificial cerebrospinal fluid (aCSF) containing 119 mM NaCl, 2.5 mM KCl, 1 mM NaH_2_PO_4_, 26.2 mM NaHCO_3_, and 11 mM glucose bubbled with 95% (*v*/*v*) O_2_ and 5% (*v*/*v*) CO_2_ to maintain pH. aCSF was supplemented with 4 mM CaCl_2_ and 4 mM MgSO_4_. Five micromolar 2-chloroadenosine was added to dampen epileptiform activity and 0.1 mM picrotoxin to block GABA (A) receptors. Osmolality was adjusted to 305–315 mOsm. Borosilicate glass recording pipettes were filled with a pipette internal solution suited for whole-cell recordings buffered at 7.3–7.4 pH and composed of the following (in mM): 135 CsMeSO_4_, 8 NaCl, 10 HEPES, 0.3 EGTA, 5 QX-314, 4 Mg-ATP, and 0.3 Na-GTP.

All electrophysiology experiments were performed on an upright Olympus BX50WI microscope. Differential interference phase contrast microscopy was used to identify untransfected DG granule neurons and CA1 pyramidal neurons, while GFP-transfected cells were identified using epifluorescence microscopy. Stratum radiatum pathway and perforant pathway afferents were stimulated with a monopolar glass electrode to produce simultaneous postsynaptic currents measured from a pair of untransfected and transfected CA1 pyramidal and DG granule neurons, respectively. This approach allows for a pairwise, internally controlled comparison of the consequences of acute genetic manipulations in the transfected postsynaptic neuron (Arnold and Heintz, 1997; McAllister, 2004; Pfeiffer et al., 2010; Rao et al., 2019). Membrane voltage was held at −70 mV to measure AMPAR-evoked excitatory postsynaptic currents (eEPSCs) and at +40 mV to measure NMDAR-eEPSCs. NMDAR current amplitudes were measured at 150 ms after stimulation to avoid contamination from AMPAR current. AMPAR- and NMDAR-mediated currents were typically recorded from the same cell. Paired-pulse facilitation was induced by delivery of two consecutive stimuli at intervals of 20 ms. No more than one pair was recorded from a single hippocampal slice. Membrane-holding current, pipette series resistance, and input resistance were monitored throughout recording sessions. Data were gathered through a MultiClamp 700B amplifier (Axon Instruments), filtered at 2 kHz, and digitized at 10 kHz.

### Acute slice preparation and LTP

For acute slice experiments, in utero electroporation was performed as previously described (Elias et al., 2008). P18–P24 mice were subjected to isoflurorane exposure and verified to be under a surgical plane of anesthesia. Three hundred micrometer transverse slices were cut from a freshly harvested hippocampus using a ZERO1 vibrating microtome (Ted Pella). Slices were collected in cold high-sucrose low–sodium cutting solution containing the following (in mM), 2.5 KCl, 0.5 CaCl_2_, 7 MgCl_2_, 1.25 NaH_2_PO_4_, 25 NaHCO_3_, 7 glucose, 210 sucrose, and 1.3 ascorbic acid, bubbled with 95% O_2_ and 5% CO_2_. Thereafter slices were transferred to aCSF but supplemented with CaCl_2_ at 2.5 mM and MgSO_4_ at 1.3 mM with osmolality adjusted to 290–295 mM. Slices were incubated in aCSF saturated with 95% O_2_ and 5% CO_2_ at 37°C and RT for 40 min each after which slices were transferred to the recording chamber maintained in oxygenated aCSF. Borosilicate recording pipettes were filled with the same internal solution as organotypic slices. To minimize runup of baseline responses during LTP, cells were held cell attached for ∼1–2 min before breaking into the cell. GFP-transfected neurons were held at −70 mV in whole-cell configuration, while a borosilicate pipette filled with aCSF was used to stimulate Schaffer collateral afferents in the stratum radiatum. Baseline individual synaptic responses were measured for ∼2 min. Thereafter, LTP was induced within 5 min of achieving the whole-cell configuration by holding neurons at 0 mV during a 2 Hz stimulation of Schaffer collateral afferents for 90 s.

### Imaging and spine analysis

Cultured hippocampal slices were transfected with pFHUGW-GFP shRNA constructs alone or pFHUGW-GFP shRNA and pCAGGS-mCherry cDNA constructs ∼18–20 h after plating using biolistic transfection. The experimenter was blinded to the genotype during subsequent processing and imaging. Slices were fixed in 4% PFA, 4% sucrose in 1× PBS, and washed with 1× PBS and then cleared with an abbreviated SeeDB-based protocol and mounted on microscope slides (Ke et al., 2013). High-resolution confocal *Z*-stacks of spine-containing CA1 pyramidal neuron apical dendrites were acquired on a Zeiss 510 microscope using a Plan Apochromat 40×/1.4 Oil DIC objective. *Z*-stacks were collected at 2,048 px × 2,048 px, 16-bit *X*-*Y* pixel dimensions, with *X*-*Y* spatial resolution of 70 nm and axial resolution of 500 nm with a 488 nm laser excitation wavelength. Automated analysis of dendritic segments and spines was performed using the commercially available software Filament Tracer (Imaris 9.1.2, Bitplane). For each cell, an ∼60 μm dendritic segment was manually selected for analysis, and thresholds for dendritic surface and spine rendering were set (10 μm dendrite length; minimum spine diameter and maximum spine length were set to 0.2 and 2 μm, respectively). Data were exported into Microsoft Excel and analyzed and graphed using R Studio (Version 1.1.153).

### Lentivirus preparation

Lentivirus was generated by transfecting HEK293T cells with plasmids pVSV-G and psPAX2 along with shRNA expressing constructs using FuGENE HD transfection reagent (Roche). Viral particle containing supernatant was collected 48 h later. Supernatant was filtered at 70 µm, concentrated using PEG-it (System Biosciences). Then viral particles were pelleted via centrifugation and resuspended in DMEM.

### Western blotting

Embryonic Day (E)16.5 rat or mouse hippocampi were dissected, dissociated, and cultured in DMEM with 10% FBS. Neurons were plated onto six-well plates and infected with 20 µl virus (pFHUG-IRES-GFP lentivirus or pFHUG-Tiam2-shRNA-IRES-GFP lentivirus). At DIV 21, cell lysates were prepared and run on a 4–15% Mini-PROTEAN TGX Precast Protein Gel (Life Technologies) with 25–50 µg of protein loaded per lane. For experiments involving the expression of constructs in HEK293T cells (ATCC), cells seeded in six-well plates were transfected with 2 µg of total DNA, and lysates were prepared after 72 h of expression. Membranes were probed with antibodies specific for Tiam2 (1:100; sc-514090, Santa Cruz Biotechnology, and ab199426, Abcam), Tiam1 (1:100; sc-393315, Santa Cruz Biotechnology), and β-actin [1:1000, (13E5) Rabbit mAb 4970, Cell Signaling Technology]. Horseradish peroxidase-conjugated secondary antibodies were used for chemiluminescent detection. Membranes were scanned using Bio-Rad ChemiDoc MP Imaging System and exported using Image Lab Software.

### Experimental design and data analysis

For all experiments, at least three male and three female rat pups were used. Imaging analysis was performed blind to genotype. All electrophysiological data are expressed as mean (*M*) ± standard error measurement (SEM). Data from simultaneous dual whole-cell recordings are shown within scatterplots, wherein each open circle represents one paired recording and a closed circle represents the average of all paired recordings. If the average data point falls above the diagonal line, it indicates that the eEPSC is lower in the control neuron and vice versa. Paired-pulse facilitation was calculated by dividing the peak response from the second stimulus by the peak response of the first stimulus.

Statistical significance was determined using Wilcoxon signed-rank test for paired dual whole-cell patch–clamp data, Wilcoxon rank-sum test for imaging data, and Student's *t* test for paired-pulse facilitation data. All *p* values <0.05 were considered significant and denoted with a single asterisk, *p* values <0.01 were denoted with a double asterisk, and *p* values <0.001 were denoted with a triple asterisk. All error bars represent standard error measurement. Sample sizes in the present study are as reported previously (Herring and Nicoll, 2016; Incontro et al., 2018).

Coefficient of variation (CV) analysis was performed by calculating the *M* and standard deviation (SD) of AMPAR-eEPSCs (Kokaia et al., 1998). At least 20 consecutively recorded current amplitudes for both control and transfected cells within a paired dual whole-cell patch–clamp recording were used to obtain the CV, calculated as SD / *M*. Theoretical and experimental work has shown that CV^−2^ (*M*^2^ / SD^2^) is invariant with changes in the quantal size (i.e., the number of AMPA receptors at all synapses) and that CV^−2^ varies predictably with changes in the quantal content (i.e., the number of functional synapses containing AMPA receptors) according to the following equation CV^−2^ = *n* × *P*_r_ / (1 − *P*_r_) where *n* is the number of vesicle release sites and *P*_r_ is the probability of presynaptic release. To observe the eEPSC variance with changes in the mean amplitude, the CV^−2^ values for transfected and control cells were plotted on the *y*-axis, and the ratios of means for transfected and control cells were plotted on the *x*-axis. Values above the 45° (*y* = *x*) line indicate increases in quantal content, while values approaching the horizontal line (*y* = 1) indicate a change in the quantal size as ultimately responsible for the difference in AMPAR-eEPSC amplitude between the control and transfected cells. For LTP, individual experiments were normalized to the baseline (before stimulation), and 12 consecutive responses were averaged to generate 1 min bins, which were then averaged to generate summary graphs. Bar graphs of LTP are averaged eEPSC values for the first 2 min (prior to LTP induction) and last 2 min of LTP summary graphs. For immunoblotting experiments, images are representative of three experimental replicates. For quantification, proteins were transferred to the same nitrocellulose membrane and probed in parallel for technical consistency. Background-adjusted band intensities were measured on Image Lab (Bio-Rad Laboratories), graphed on GraphPad Prism, and analyzed for statistical significance using the Mann–Whitney test.

## Results

### Tiam2 knockdown produces significant reductions in AMPAR- and NMDAR-mediated neurotransmission at CA1 pyramidal neurons

Immunostaining of rat hippocampal slices revealed robust Tiam2 expression throughout the hippocampal formation (Fig. 1*A*; Extended Data Fig. 1-1*A*) consistent with reports of Tiam2 mRNA expression (Zhao et al., 2001; Lein et al., 2007; Cembrowski et al., 2016). To determine whether Tiam2 regulates glutamatergic neurotransmission in CA1 pyramidal neurons, we utilized a shRNA (Goto et al., 2011) that depletes Tiam2 protein expression (Fig. 1*B*; Extended Data Fig. 1-1*B*). We biolistically transfected rat hippocampal slices with Tiam2 shRNA and simultaneously recorded currents in transfected and untransfected CA1 pyramidal neurons evoked by Schaffer collateral stimulation (Fig. 1*C*). Our measurements of AMPAR- and NMDAR-mediated eEPSCs revealed a substantial ∼50 and ∼40% reduction in AMPAR-eEPSCs and in NMDAR-eEPSCs amplitudes, respectively (Fig. 1*D*,*E*). This is in contrast to the homolog Tiam1, whose depletion produced no observable effect of glutamatergic neurotransmission in CA1 pyramidal neurons (Rao et al., 2019).

To examine whether Tiam2's impact on glutamatergic neurotransmission is the result of presynaptic mechanism, we performed an analysis of paired-pulse facilitation at Schaffer collateral→CA1 pyramidal neuron synapses. We found that loss of Tiam2 protein expression in CA1 pyramidal neurons had no effect on paired-pulse facilitation (Fig. 1*F*), indicating presynaptic neurotransmitter release mechanisms are unaltered and Tiam2's role in supporting glutamatergic synapse function is postsynaptic.

### Tiam2 knockdown reduces spine density in CA1 pyramidal neurons

Next, we determined the synaptic alteration underlying reductions in AMPAR- and NMDAR-eEPSC amplitudes following Tiam2 knockdown. These alterations could arise from uniform reduction in postsynaptic AMPARs and NMDARs across all glutamatergic synapses or loss of a subset of functional glutamatergic synapses in CA1 pyramidal neurons. We performed CV^−2^ analysis, with a comparison of the normalized variance of eEPSC amplitudes from two neurons receiving the same stimulus to identify changes in the quantal size and/or quantal content (Del Castillo and Katz, 1954; Bekkers and Stevens, 1990; Malinow and Tsien, 1990; Gray et al., 2011; Levy et al., 2015). Data points that lie on the horizontal dotted line indicate changes in the quantal size and represent uniform changes in receptor function across existing synapses. Data points that fall on or near the diagonal dotted line indicate changes in the quantal content and represent changes in the number of functional glutamatergic synapses (Bekkers and Stevens, 1990; Malinow and Tsien, 1990). The averages for both AMPAR and NMDAR excitatory currents fell on or near the diagonal dotted line (Fig. 1*G*,*H*), showing that deficits in current amplitudes are explained by changes in quantal content. Thus, reductions in AMPAR- and NMDAR-eEPSC amplitude following Tiam2 knockdown result from a reduced number of functional glutamatergic synapses.

**Figure 2.**
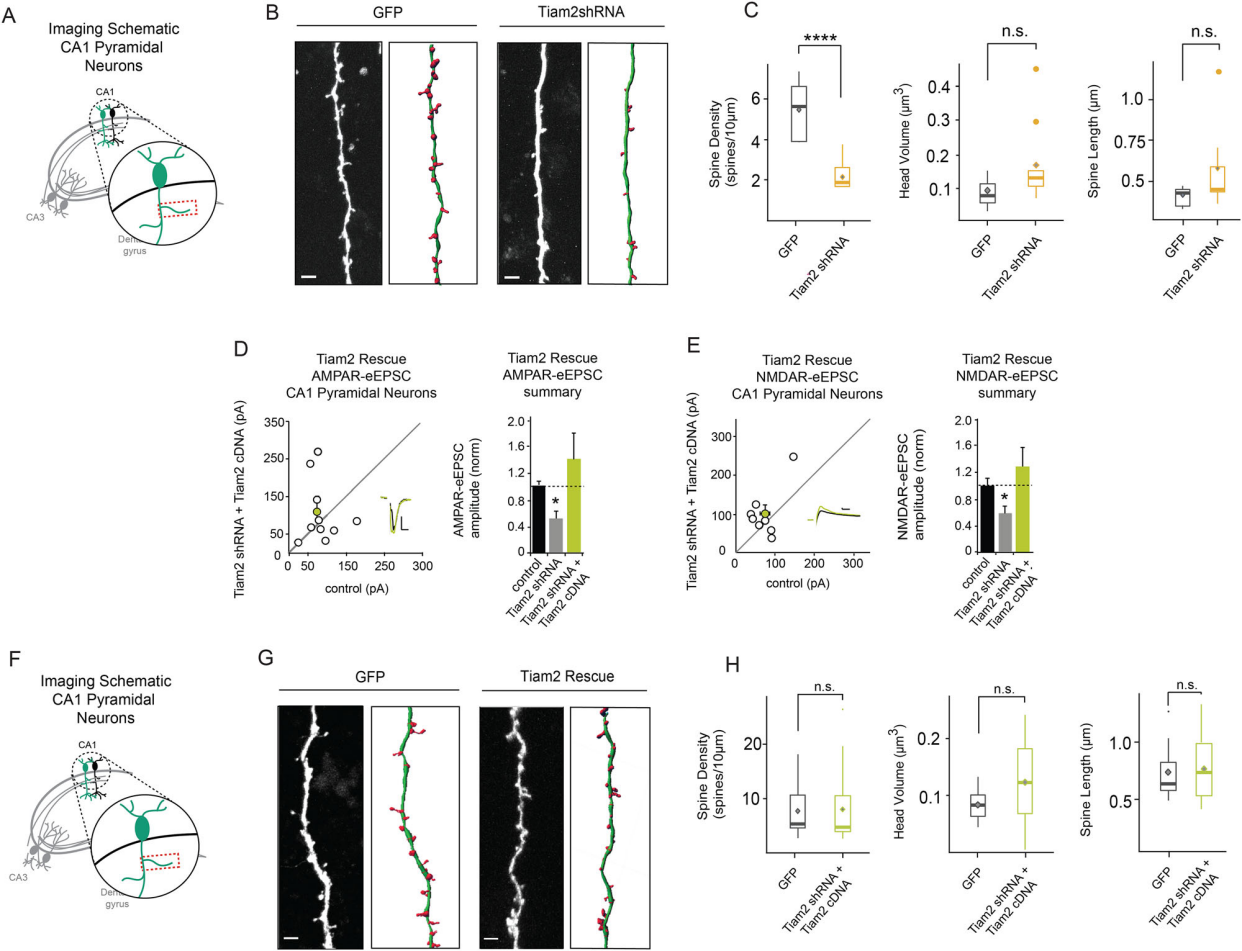
Tiam2 depletion results in reduced spine density in CA1 pyramidal neurons. ***A***, Schematic of areas of image acquisition from CA1 pyramidal neuron dendrites. ***B***, ***G***, Representative dendritic segments and corresponding reconstructed filaments (scale bars: 4 μm). ***C***, Boxplots show spine density is reduced in CA1 pyramidal neurons expressing Tiam2 shRNA (yellow) compared with GFP-expressing control neurons (gray; *p* = 0.0000411 for spine density; *p* = 0.05031 for spine head volume; *p* = 0.22 for spine length; for GFP *n* = 9 segments, *n* = 6 cells; for Tiam2 shRNA *n* = 9 segments, *n* = 9 cells; Wilcoxon rank-sum test). ***D***, ***E***, Scatterplots with AMPAR- and NMDAR-eEPSC amplitudes, respectively, plotted against paired control neuron eEPSCs (open circles) with *M* ± SEM (filled circles). (Insets) Representative current traces from control (black) and transfected (yellow) neurons with stimulation artifacts removed (scale bars: 20 pA for both AMPAR-eEPSCs and NMDAR-eEPSCs, 20 ms for AMPAR, 50 ms for NMDAR). Barplots show average AMPAR and NMDAR-eEPSC amplitudes (±SEM) of transfected CA1 pyramidal neurons normalized to respective control cell average eEPSC amplitudes (black). Tiam2 cDNA expression restores AMPAR- and NMDAR-eEPSC amplitude in CA1 pyramidal neurons coexpressing Tiam2 shRNA (for AMPAR-eEPSCs *p* = 0.4; *n* = 9 pairs; for NMDAR-eEPSC *p* = 0.2; *n* = 8 pairs; Wilcoxon signed-rank test). ***F***, Schematic of areas of image acquisition from CA1 pyramidal neuron dendrites. ***G***, ***H***, No significant differences were detected in any spine parameters in Tiam2 shRNA and Tiam2 cDNA coexpressing spines in CA1 pyramidal neurons compared with corresponding GFP-expressing control neurons. ***H***, Boxplots show unaltered spine density in CA1 pyramidal neurons expressing Tiam2 cDNA and Tiam2 shRNA (green) compared with GFP-expressing control neurons (gray boxes; *p* = 0.7413 for spine density; *p* = 0.7376 for spine head volume; *p* = 0.5681 for spine length; for GFP *n* = 12 segments, *n* = 5 cells; for Tiam2 shRNA *n* = 16 segments, *n* = 11 cells; Wilcoxon rank-sum test). ****p < 0.0001. See Extended Data Figure 2-1 for more details.

Tiam2 catalyzes small-GTPase Rac1 activity, which promotes actin-dependent formation of dendritic spines (Tashiro and Yuste, 2004; Goto et al., 2011). Similar reductions in AMPAR- and NMDAR-eEPSC amplitudes and CV^−2^ analysis suggest an underlying reduction in dendritic spine density. We compared Tiam2 shRNA- and GFP-transfected CA1 pyramidal neurons (Fig. 2*A*) and observed a dramatic loss in the spine number relative to control neurons (Fig. 2*B*,*C*). Spine length and other parameters remained unchanged (Fig. 2*C*; Extended Data Fig. 2-1*A*–*D*). We verified that reduced AMPAR and NMDAR function produced by Tiam2 knockdown could be rescued by recombinant Tiam2 expression. To do this we coexpressed Tiam2 shRNA and a recombinant shRNA-resistant Tiam2 cDNA in CA1 pyramidal neurons and recorded AMPAR- and NMDAR-eEPSCs. We found that AMPAR- and NMDAR-eEPSC amplitudes were similar between control and transfected neurons, demonstrating Tiam2 cDNA was sufficient to rescue Tiam2 knockdown (Fig. 2*D*,*E*). The expression of Tiam2 cDNA was also sufficient to rescue deficits in spine density in CA1 pyramidal neuron dendrites (Fig. 2*F*–*H*; Extended Data Fig. 2-1*E*–*H*). These results further demonstrate that Tiam2 shRNA is specific for endogenous Tiam2, and the loss-of-function phenotypes we observe are a result of targeted reduction in Tiam2 protein expression. Moreover, the reduction in dendritic spine density corroborates our previous functional analyses and is consistent with Tiam2's role in supporting glutamatergic synapse structure and function in CA1 pyramidal neurons.

10.1523/ENEURO.0500-21.2024.f2-1 Figure 2-1 **Spine imaging and rescue of Tiam2 depletion in CA1 pyramidal neurons A-H** No significant differences were detected in other spine parameters in Tiam2 knockdown or Tiam2 rescue in CA1 pyramidal neurons compared with respective GFP-expressing control neurons. Boxplots show spine parameters for CA1 pyramidal neurons transfected with Tiam2 shRNA (yellow) or Tiam2 shRNA and Tiam2 cDNA (green) compared to GFP expressing control neurons (grey) (*for Tiam2 knockdown*: for GFP n = 9 segments, n = 6 cells, for Tiam2 shRNA n = 9 segments, n = 9 cells, p = 0.06253 for Spine Neck Diameter, p = 0.01876 for Spine Neck Volume, p = 0.06253 for Spine Diameter, p = 0.1359 for Spine Volume; *for Tiam2 rescue*: GFP n = 12 segments, n = 5 cells, Tiam2 shRNA and Tiam2 cDNA n = 16 segments, n = 11 cells, p = 0.2098 for Spine Neck Diameter, p = 0.1894 for Spine Neck Volume, p = 0.1894 for Spine Diameter, p = 0.3758 for Spine Volume; Wilcoxon rank-sum test). Download Figure 2-1, TIF file.

### Tiam2 has a DH1 domain-dependent role in neurotransmission at Schaffer collateral–CA1 pyramidal neurons and is dispensable for baseline neurotransmission at perforant path–DG granule neurons

**Figure 3.**
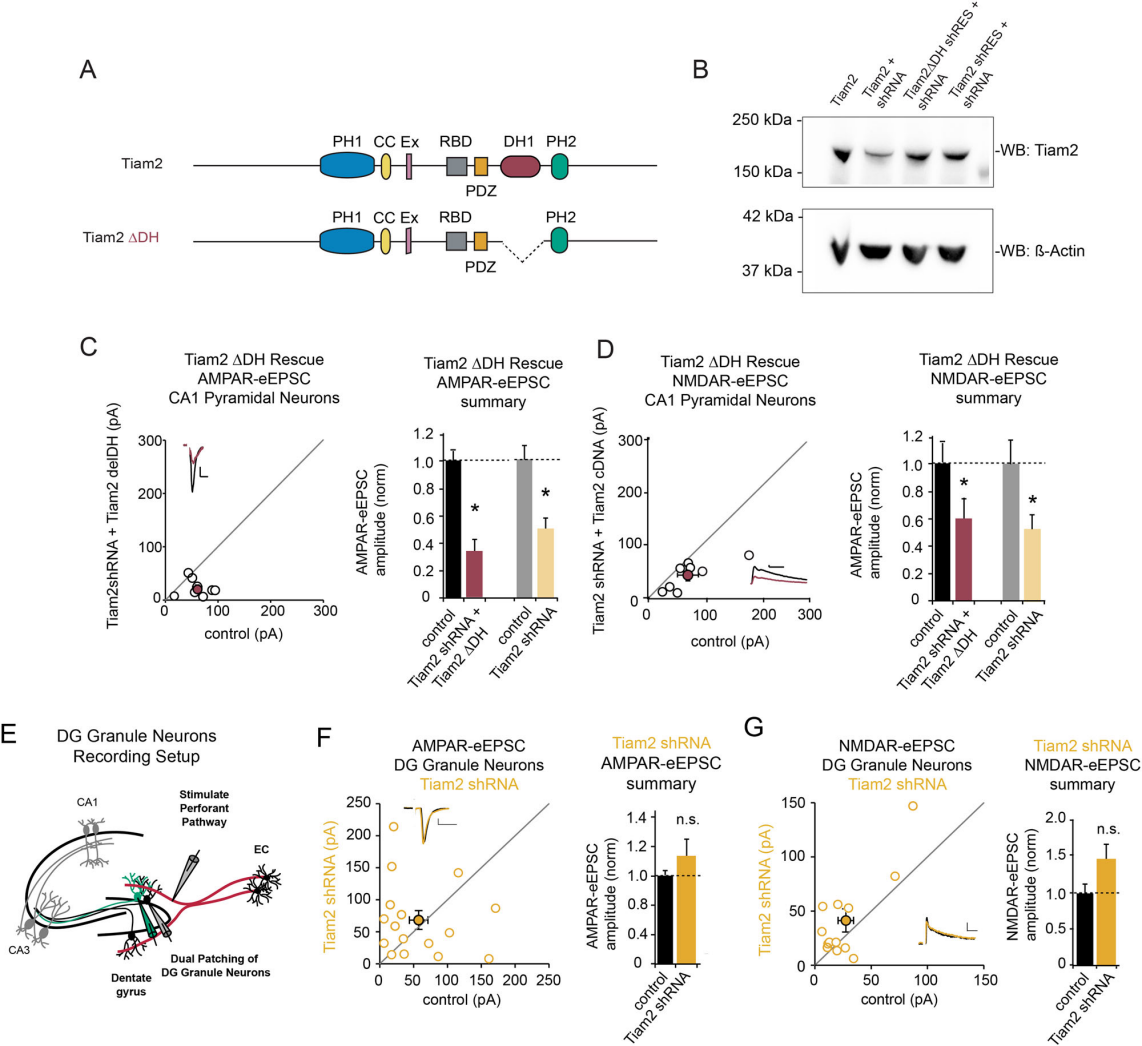
Tiam2 depletion is not rescued by DH1 domain-lacking Tiam2 in CA1 pyramidal neurons and has no effect on neurotransmission in DG granule neurons. ***A***, Illustration of Tiam2's protein domains; full-length Tiam2 (upper) and Tiam1 ΔDH (lower). ***B***, Immunoblot of shRNA-resistant Tiam2 ΔDH and Tiam2 cDNA coexpressed with Tiam2 shRNA in HEK293 cells. ***C***, ***D***, ***F***, ***G***, Scatterplots of AMPAR- and NMDAR-eEPSC amplitudes for CA1 pyramidal neurons expressing Tiam2 shRNA (yellow) and coexpressing Tiam2 shRNA and Tiam2 ΔDH (red), respectively, plotted against paired control neuron eEPSCs (open circles) with *M* ± SEM (filled circles). (Inset) Representative current traces from control (black) and transfected (yellow or red) neurons stimulation artifacts removed (scale bars: 20 pA for AMPA, 20 ms for AMPA). Barplots show average AMPAR and NMDAR-eEPSC amplitudes (±SEM) of transfected (yellow or red) CA1 pyramidal neurons normalized to respective control cell average eEPSC amplitudes (black). ***C***, ***D***, Tiam2 ΔDH does not restore AMPAR- and NMDAR-eEPSC amplitude in CA1 pyramidal neurons coexpressing Tiam2 shRNA (for AMPAR-eEPSCs *p* = 0.016, *n* = 8 pairs; for NMDAR-eEPSC *p* = 0.016, *n* = 7 pairs; Wilcoxon signed-rank test.). ***E***, Schematic representation of electrophysiological recording setup for DG granule neurons. ***F***, ***G***, Tiam2 knockdown has no effect on AMPAR- and NMDAR-eEPSC amplitude in DG granule neurons (for AMPAR-eEPSCs *p* = 0.67, *n* = 16 pairs; for NMDAR-eEPSC *p* = 0.08, *n* = 13 pairs; Wilcoxon signed-rank test) *p < 0.05, n.s., not significant. See Extended Data Figure 3-1 for more details.

The DH1 domain mediates actin polymerization via Rac1 (Fig. 3*A*; Zheng et al., 1996; Worthylake et al., 2000; Karnoub et al., 2001). We verified that DH1 domain-lacking recombinant Tiam2 ΔDH (Fig. 3*A*) has expression comparable to wild-type Tiam2 in HEK293T cells (Fig. 3*B*). We found that CA1 pyramidal neurons expressing Tiam2 ΔDH and Tiam2 shRNA showed a ∼60 and ∼40% reduction in AMPAR- and NMDAR-eEPSC amplitudes, respectively (Fig. 3*C,D*), which is comparable to reductions resulting from shRNA-mediated Tiam2 knockdown. These data confirm that the DH1 domain is necessary for Tiam2's role in neurotransmission at Schaffer collateral–CA1 pyramidal neurons.

10.1523/ENEURO.0500-21.2024.f3-1 Figure 3-1 **Tiam2 depletion has no effect on spine parameters in DG granule neurons A** Schematic of areas of image acquisition from CA1 pyramidal neuron dendrites. **E, F** Boxplots show spine parameters for DG granule neurons transfected with Tiam2 shRNA (yellow) or GFP expressing control neurons (grey) (*for Tiam2 knockdown*: for GFP n = 16 segments, n = 8 cells, for Tiam2 shRNA n = 15 segments, n = 10 cells, p = 0.3599 for Spine Density, p = 0.6965 for Spine Head Volume, p = 0.1728 for Spine Length, p = 0.7618 for Spine Neck Diameter, p = 0.6334 for Spine Neck Volume, p = 0.9654 for Spine Diameter, p = 0.4598 for Spine Volume; Wilcoxon rank-sum test). n.s. – not significant. Download Figure 3-1, TIF file.

Tiam2 has significant expression in DG granule neurons (Fig. 1*A*). Surprisingly, we found that Tiam2 depletion in DG granule neurons had no effect on AMPAR and NMDAR current amplitude (Fig. 3*F,G*), with no alterations in spine morphology (Extended Data Fig. 3-1*A–C*). This suggests a DH1-dependent role of Tiam2 at Schaffer collateral→CA1 pyramidal neurons and no role for Tiam2 in neurotransmission at perforant path→DG granule neuron synapses.

### Tiam1 and Tiam2 are individually dispensable for functional LTP in CA1 pyramidal neurons

**Figure 4.**
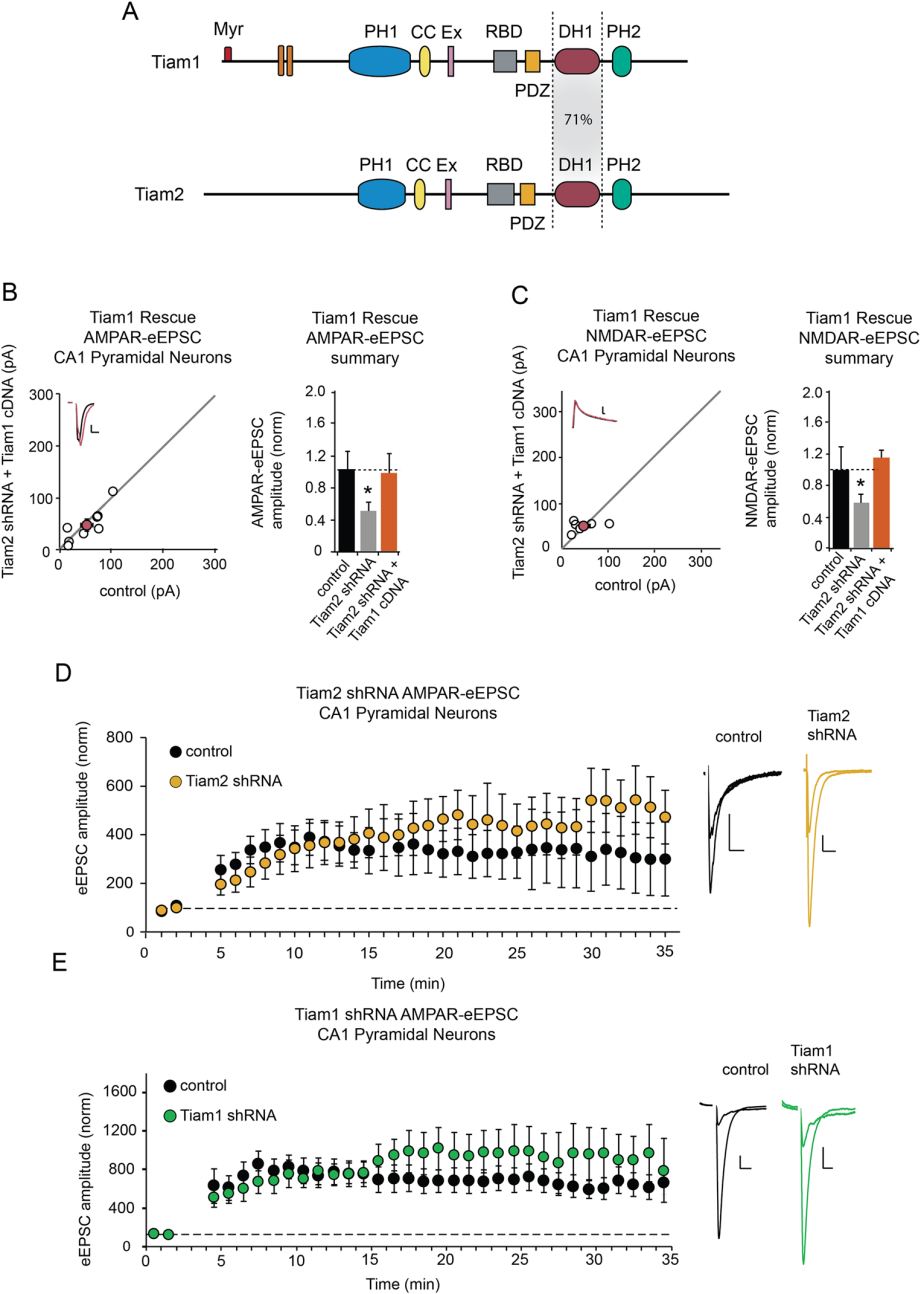
Tiam1 and Tiam2 are dispensable for functional LTP in CA1 pyramidal neurons. ***A***, Illustration of Tiam1 and Tiam2 protein domains; full-length Tiam1 (upper) and Tiam2 (lower). ***B***, ***C***, Scatterplots with AMPAR- and NMDAR-eEPSC amplitudes for CA1 pyramidal neurons coexpressing Tiam2 shRNA and Tiam1 cDNA (orange), respectively, plotted against paired control neuron eEPSCs (open circles) with corresponding *M* ± SEM (filled circles). (Inset) Representative current traces from control (black) and transfected (green) neurons stimulation artifacts removed (scale bars: 20 pA for AMPA, 20 ms for AMPA). Barplots show average AMPAR and NMDAR-eEPSC amplitudes (±SEM) of CA1 pyramidal neurons normalized to respective control cell average eEPSC amplitudes (black bar). ***B***, ***C***, Tiam1 cDNA expression restores AMPAR- and NMDAR-eEPSC amplitude in CA1 pyramidal neurons coexpressing Tiam2 shRNA (for AMPAR-eEPSCs *p* = 0.5, *n* = 8 pairs; for NMDAR-eEPSC *p* = 0.8, *n* = 6 pairs, Wilcoxon signed-rank test; insets). ***D***, ***E***, LTP induction is unaltered by Tiam1 or Tiam2 depletion in CA1 pyramidal neurons. Plots of *M* ± SEM AMPAR-eEPSC amplitude of control untransfected CA1 pyramidal neurons (black) and CA1 pyramidal neurons electroporated with Tiam1shRNA (green) or Tiam2shRNA (yellow) normalized to the *M* AMPAR-eEPSC amplitude before an LTP induction protocol (arrow; Tiam1: control, *n* = 9 neurons; Tiam1shRNA, *n* = 7 neurons; Tiam2: control, *n* = 9 neurons; Tiam2 shRNA, *n* = 7 neurons). Sample AMPAR-eEPSC current traces from control (black) and electroporated (green or yellow) neurons before and after LTP induction are shown to the right of the graphs. (Scale bars: 20 ms, 20 pA.) *p < 0.05, Wilcoxon signed-rank test. See Extended Data Figure 4-1 for more details.

Although Tiam1 and Tiam2 have similar domains, they display distinct ligand specificities in vitro (Shepherd et al., 2011). Tiam2 shares 71% of DH1 domain identity with Tiam1 (Fig. 4*A*; Chiu et al., 1999; Hoshino et al., 1999; Cook et al., 2014). Prior evidence indicates Tiam1 expression is detected in DG granule neurons, which we confirmed (Extended Data Fig. 4-1*A*; Rao et al., 2019; Cheng et al., 2021). We wondered if Tiam1 could substitute for Tiam2 in CA1 pyramidal neurons. Neurons coexpressing Tiam2 shRNA and Tiam1 cDNA had glutamatergic neurotransmission comparable with control neurons (Fig. 4*B*,*C*), indicating that Tiam1 can substitute for Tiam2 function in neurotransmission at Schaffer collateral→CA1 pyramidal neuron synapses.

10.1523/ENEURO.0500-21.2024.f4-1 Figure 4-1 **A Tiam1 antibody validation** Tiam1 immunolabelling (white) in whole hippocampal slice co-localized with DG granule neuron specific Prox1 (red) and DAPI (blue), (inset) DG granule cell body layer (scale bar: 500μm). **B** Western blot showing shRNA-mediated reduction of Tiam1 and unaltered Tiam2 expression in Tiam1 shRNA expressing mouse hippocampal neurons, **C** Western blot showing shRNA-mediated reduction of Tiam2 and unaltered Tiam1 expression in Tiam2 shRNA expressing mouse hippocampal neurons. Barplots show mean ± SEM of experimental replicates (Tiam1 shRNA: Tiam1 n = 5, Tiam2 n = 4, Tiam2 shRNA: Tiam2 n = 4, Tiam1 n = 3, ****p < 0.0001, Mann- Whitney test). Download Figure 4-1, TIF file.

Having established Tiam2 is necessary for normal baseline neurotransmission at CA1 pyramidal neuron synapses; we tested whether Tiam2 affects mechanisms of synaptic plasticity. In acute mouse hippocampal slices where Tiam2 shRNA was delivered via in utero electroporation, we found Tiam2 depletion has no effect on the induction or maintenance of functional LTP at Schaffer collateral–CA1 pyramidal neurons (Fig. 4*D*). It is possible this lack of effect was due to a compensatory increase in Tiam1, but shRNA-mediated depletion does not affect expression of the either homolog in vitro (Extended Data Fig. 4-1*B*,*C*). Similarly, we found that Tiam1 knockdown produced no significant effect on LTP induction or maintenance in CA1 pyramidal neurons (Fig. 4*E*), suggesting that neither Tiam2 nor Tiam1 individually plays an essential role in this form of synaptic plasticity at Schaffer collateral→CA1 pyramidal synapses.

## Discussion

Loss-of-function studies describe the contribution of differential gene expression towards input-specific regulation of synaptic transmission and plasticity (Arnold and Heintz, 1997; Elias et al., 2008; Westerink et al., 2012). Here, we found the RhoGEF Tiam2 is essential for normal glutamatergic neurotransmission at Schaffer collateral→CA1 pyramidal neuron synapses. We show Tiam2 depletion in CA1 pyramidal neurons produces diminished synaptic currents conducted by postsynaptic glutamate receptors and that this depletion depends on the catalytic DH1 domain. Furthermore, Tiam2 depletion does not affect presynaptic glutamate release and reduces dendritic spine density, indicating that Tiam2 regulation of synapses is restricted to the postsynaptic compartment. Prior evidence indicates endogenous Tiam1 depletion does not affect baseline neurotransmission at Schaffer collateral→CA1 pyramidal neuron synapses (Rao et al., 2019). While we found recombinant Tiam1 rescues deficits from Tiam2 depletion at these synapses, differential ligand binding within other domains, PDZ or Pleckstrin homology coiled-coil extension (Zheng et al., 1996; Shepherd et al., 2011; Rao et al., 2019), may confer functional differences at other synapses. Tiam2 expression is evident within other hippocampal subregions. Surprisingly, Tiam2 depletion at perforant path–DG granule neurons produced no alterations in synaptic transmission. Overall, our analyses are consistent with necessary role of Tiam2 in the regulation of glutamatergic synapses in CA1 pyramidal neurons.

Several studies show Tiam1 is highly expressed in DG granule neurons (Ehler et al., 1997; Zhao et al., 2001; Lein et al., 2004, 2007; Rao et al., 2019; Cheng et al., 2021). This is consistent with our immunostaining of hippocampal slices (Extended Data Fig. 4-1*A*); however there are reports of Tiam1 expression in CA1 as well (Sasaki et al., 2010). We cannot rule out that Tiam1 plays a functional role in CA1 pyramidal neurons, but we find Tiam1 and Tiam2 are dispensable for functional LTP at Schaffer collateral→CA1 pyramidal neuron synapses. RhoGEFs Trio and Kalirin are required for the expression of LTP in CA1 pyramidal neurons (Herring and Nicoll, 2016). Since our experimental paradigm produces a depletion and does not eliminate protein expression, it is entirely possible that low levels of Tiam1 or Tiam2 protein, together with Trio and Kalirin, contribute to LTP in CA1 pyramidal neurons. Recent studies report a Tiam1-mediated deficit in structural synaptic plasticity in CA1 pyramidal neurons (Kojima et al., 2019; Saneyoshi et al., 2019), and structural and functional LTP are well correlated (Matsuzaki et al., 2004). Our data support a dispensable role for Tiam1 and Tiam2 in LTP. This discrepancy may be explained by a partial contribution to plasticity by Tiam1, a possible cell autonomous role, or a role for Tiam1 at entorhinal→CA1 pyramidal neuron synapses.

Members of the RhoGEF protein family are implicated in glutamatergic synaptic deficits associated with neurodevelopmental disorders (Volk et al., 2015; Katrancha et al., 2017), and recent exome-sequencing studies have identified autism and schizophrenia-associated de novo missense mutations in Tiam1 and Tiam2 (Need et al., 2012; Takata et al., 2013; S. Lu et al., 2022). We show that Tiam2 is critical for glutamatergic synapse function at Schaffer collateral–CA1 pyramidal neurons. Additional RhoGEF proteins, such as Kalirin, Trio, and β-PIX, have been shown to have similar roles in supporting glutamatergic synapses in these neurons (Herring and Nicoll, 2016). This, along with the data from this study, suggests that Tiam2, Trio, and Kalirin form a cohort of RhoGEFs that are together responsible for normal glutamatergic neurotransmission at CA1 pyramidal neuron synapses. An improved understanding of their role in synapse development and function could contribute to therapeutics for neurodevelopmental disorders.

## Synthesis

Reviewing Editor: Lindsay De Biase, University of California Los Angeles Decisions are customarily a result of the Reviewing Editor and the peer reviewers coming together and discussing their recommendations until a consensus is reached. When revisions are invited, a fact-based synthesis statement explaining their decision and outlining what is needed to prepare a revision will be listed below. The following reviewer(s) agreed to reveal their identity: Elena Marcello. Note: If this manuscript was transferred from JNeurosci and a decision was made to accept the manuscript without peer review, a brief statement to this effect will instead be what is listed below.

Synthesis of Reviews:

Both reviewers found the findings interesting given the lack of information on the function of the RhoGEF Tiam2 currently available, but raised substantial concerns that should be addressed before the work can be considered for publication (see specific comments below). In particular Reviewer # 1 suggested that key control experiments are needed to validate the manuscript claims.

Reviewer#1:

This work addresses the functions of the RhoGEF, Tiam2 on mediating excitatory neurotransmission, dendritic spine morphology, and synaptic plasticity (LTP) in the CA1 region of hippocampus. Previously, the authors described a function for Tiam1 in regulating excitatory neurotransmission in hippocampal DG. Overall, the findings are interesting and provide insight into the functions of this RhoGEF in neurotransmission for the first time, however the research provides an incremental advancement. Moreover, there are a series of missing controls that should be included to validate the authors' findings.

It is well established that the introduction of shRNA in of itself can alter excitatory neurotransmission and dendritic spine densities (see Alvarez_et_al, PMID: 16870727). Thus, an essential control for the AMPAR- and NMDAR-eEPSC experiments in Figure 1 would be to conduct dual patching of CA1 pyramidal neurons that have been biolistically transfected with a shRNA scramble control. Moreover, the control conditions in Figures 2D, 2E, 3B, 3C, 3D, and 3E need to be normalized and statistically analyzed in a manner that retains the error in the control group, similar to what was done in Figures 4B and 4C.

Although Tiam1 levels have been suggested to be negligible in CA1 neurons and previous work from the authors shows no effect of Tiam1 knockdown on baseline excitatory synaptic transmission, it is possible that Tiam2 shRNA may increase Tiam1 or residually low levels of Tiam1 may compensate for the loss of Tiam2 for regulating LTP. Moreover, the authors' claim regarding Tiam1 levels in CA1 can be highly contested based on a recent publication that shows ample expression of Tiam1 in CA1 hippocampal neurons (see Sasaki_et_al, PMID: 20654717, Figure 3C) and from the authors' referenced paper by Ehler_et_al (see Figure 3G). To control for this possibility of redundancy, it is essential to knock both Tiam1 and Tiam2 down in CA1 neurons and measure LTP. Moreover, given this is a different system for expressing shRNA, it is essential to demonstrate effective knockdown of Tiam1/2 and GFP expression in CA1 cells in this specific system. Finally, the discussion surrounding Tiam1 expression in CA1 needs to be corrected to accurately reflect current literature. Comparing and contrasting findings based on what has been established in the field should be highlighted in the Discussion. For example, a description of potential cell autonomous and nonautonomous features of Tiam loss would potentially reconcile discrepancies in findings and distinctions between sLTP and LTP would also benefit.

Regarding the lack of LTP phenotype, is expression of Tiam1 and Tiam2 developmentally regulated in a region-specific manner in hippocampus? This could also explain discrepancies in its expression between labs.

Assessements of dendritic spine density changes in Tiam2 shRNA experiments where the control group is only GFP is inappropriate. One must counter a control group using a shRNA scramble control. Moreover, it is unclear how many neurons were used for each group in this experiment.

Does re-expression of Tiam2 WT and mutant also rescue reductions in dendritic spine density?

The lack of phenotype observed following Tiam1 knockdown in CA1 is interesting, however it would be meaningful to perform Tiam2 knockdown studies in DG to determine if they also produce changes in excitatory neurotransmission. This would help to resolve the select region-specific functions of these two structurally similar RhoGEFs in hippocampus.

The authors need to validate that their Tiam2 cDNAs are indeed shRNA resistant.

The labels in Figures 4B and 4C below the graphs need to be corrected.

Figure 2A, PPR traces are difficult to see when superimposed and it appears that the Tiam2 shRNA group may contain multiple peaks? Displaying these separately would allow a more qualitative assessment of PPR traces.

Reviewer#2:

In this manuscript, Rao and Herrings characterized the role of the RhoGEF Tiam2 in the synapses of CA1 pyramidal neurons. Tiam2 effect of glutamatergic transmission depends on the guanine nucleotide exchange factor (GEF) domain.

Even though Tiam2 is essential for normal glutamatergic neurotransmission, neither Tiam2 nor the homolog Tiam1 are required for long-term potentiation in CA1 pyramidal neurons.

The article is well written and relevant for the identification of the molecular mechanisms controlling hippocampal synaptic function. I have only few concerns and suggestions to strengthen the data reported in the paper.

1. In addition to the Coefficient Variation analysis of NMDARs and AMPARs in control an TIAM2 shRNA expressing neurons (fig.2), I suggest using another technique, such as immunohistochemistry, to support the results of the reduction of functional synapses.

2. The authors analyzed the spines density, head volume, neck length and spine length (Fig.2). It would be important to assess the percentage of the spine categories (mushroom, thin, stubby and filopodia) to determine which spine subpopulation is affected by the lack of Tiam2.

3. Considering that the authors performed rescue experiments co-expressing Tiam2 shRNA together with shRNA resistant-Tiam2 wild-type and mutant construct and Tiam1 cDNA (Fig. 3-4), they should perform co-transfection assays to demonstrate all the constructs are similarly expressed in presence of the Tiam2 shRNA.

4. Line 203: "in" is repeated twice.
