# Supplementary figures and images for: RhoGEF Tiam2 Regulates Glutamatergic Synaptic Transmission in Hippocampal CA1 Pyramidal Neurons
Source: eNeuro. 2024 Jul 16;11(7):ENEURO.0500-21.2024. doi: 10.1523/ENEURO.0500-21.2024 (PMC11262554; doi:10.1523/ENEURO.0500-21.2024)

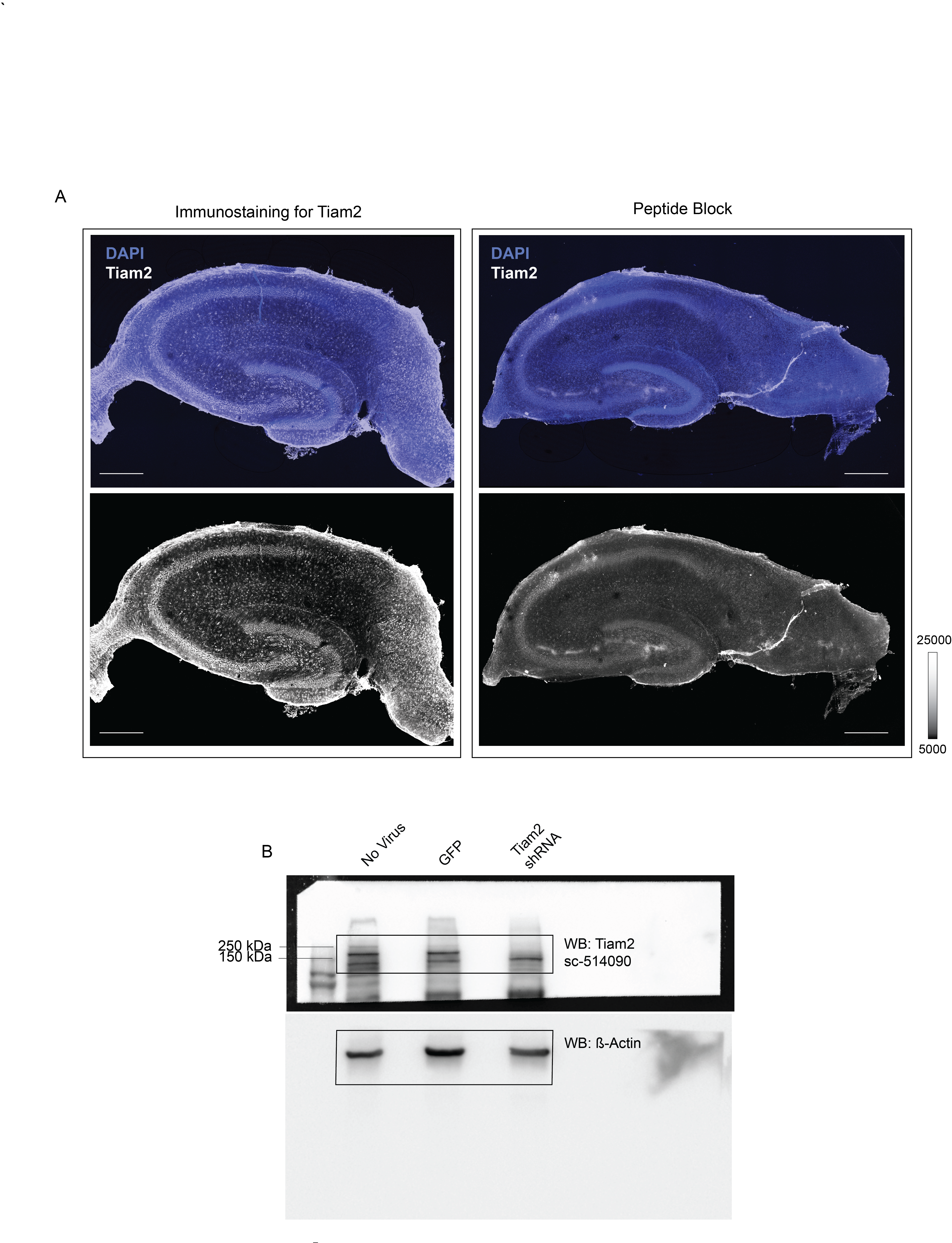

Supplement: Figure 1-1 — Antibody and shRNA validation A Tiam1 immunolabelling (white) in whole hippocampal slice co-localized with DAPI (blue), peptide control shows specificity of Tiam2 antibody (right panel) (scale bar: 500μm). B Immunoblot of DIV 21 rat hippocampal lysate transduced with no virus, GFP- and Tiam2 shRNA- expressing virus probed with Tiam2 antibody and actin (control). Download Figure 1-1, TIF file. [file eneuro-11-ENEURO.0500-21.2024-s002.tif]

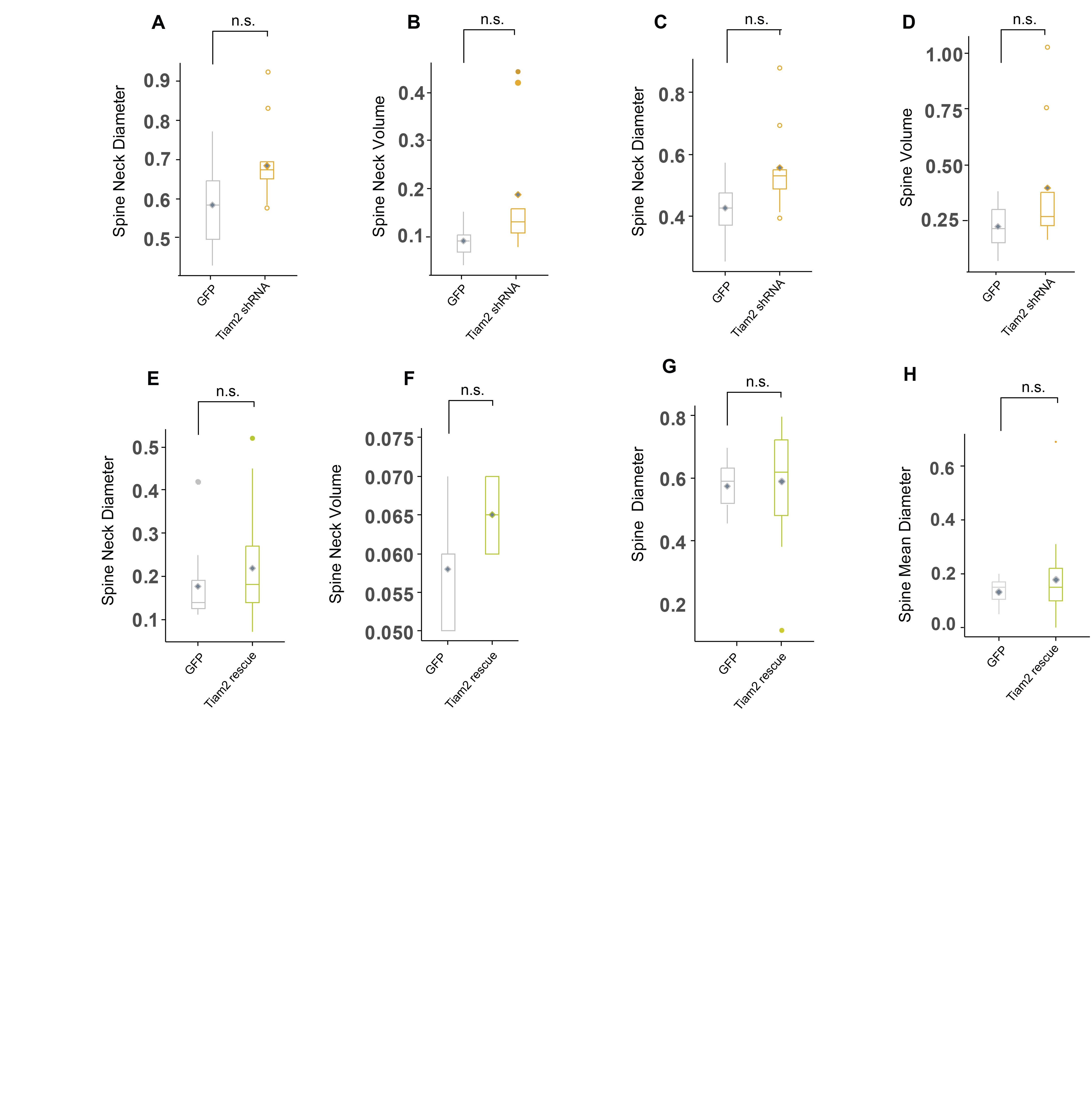

Supplement: Figure 2-1 — Spine imaging and rescue of Tiam2 depletion in CA1 pyramidal neurons A-H No significant differences were detected in other spine parameters in Tiam2 knockdown or Tiam2 rescue in CA1 pyramidal neurons compared with respective GFP-expressing control neurons. Boxplots show spine parameters for CA1 pyramidal neurons transfected with Tiam2 shRNA (yellow) or Tiam2 shRNA and Tiam2 cDNA (green) compared to GFP expressing control neurons (grey) (for Tiam2 knockdown: for GFP n = 9 segments, n = 6 cells, for Tiam2 shRNA n = 9 segments, n = 9 cells, p = 0.06253 for Spine Neck Diameter, p = 0.01876 for Spine Neck Volume, p = 0.06253 for Spine Diameter, p = 0.1359 for Spine Volume; for Tiam2 rescue: GFP n = 12 segments, n = 5 cells, Tiam2 shRNA and Tiam2 cDNA n = 16 segments, n = 11 cells, p = 0.2098 for Spine Neck Diameter, p = 0.1894 for Spine Neck Volume, p = 0.1894 for Spine Diameter, p = 0.3758 for Spine Volume; Wilcoxon rank-sum test). Download Figure 2-1, TIF file. [file eneuro-11-ENEURO.0500-21.2024-s001.tif]

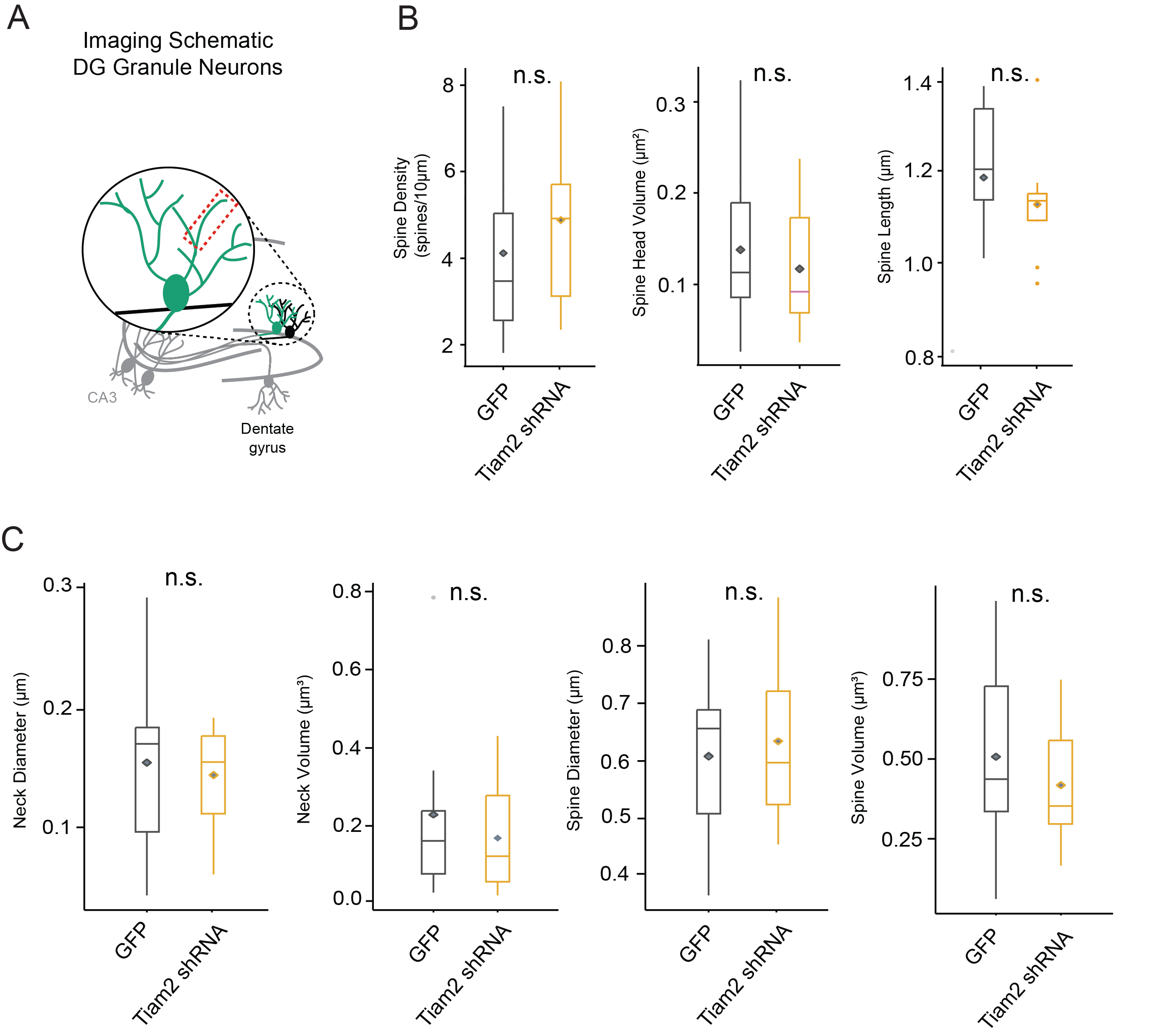

Supplement: Figure 3-1 — Tiam2 depletion has no effect on spine parameters in DG granule neurons A Schematic of areas of image acquisition from CA1 pyramidal neuron dendrites. E, F Boxplots show spine parameters for DG granule neurons transfected with Tiam2 shRNA (yellow) or GFP expressing control neurons (grey) (for Tiam2 knockdown: for GFP n = 16 segments, n = 8 cells, for Tiam2 shRNA n = 15 segments, n = 10 cells, p = 0.3599 for Spine Density, p = 0.6965 for Spine Head Volume, p = 0.1728 for Spine Length, p = 0.7618 for Spine Neck Diameter, p = 0.6334 for Spine Neck Volume, p = 0.9654 for Spine Diameter, p = 0.4598 for Spine Volume; Wilcoxon rank-sum test). n.s. – not significant. Download Figure 3-1, TIF file. [file eneuro-11-ENEURO.0500-21.2024-s003.tif]

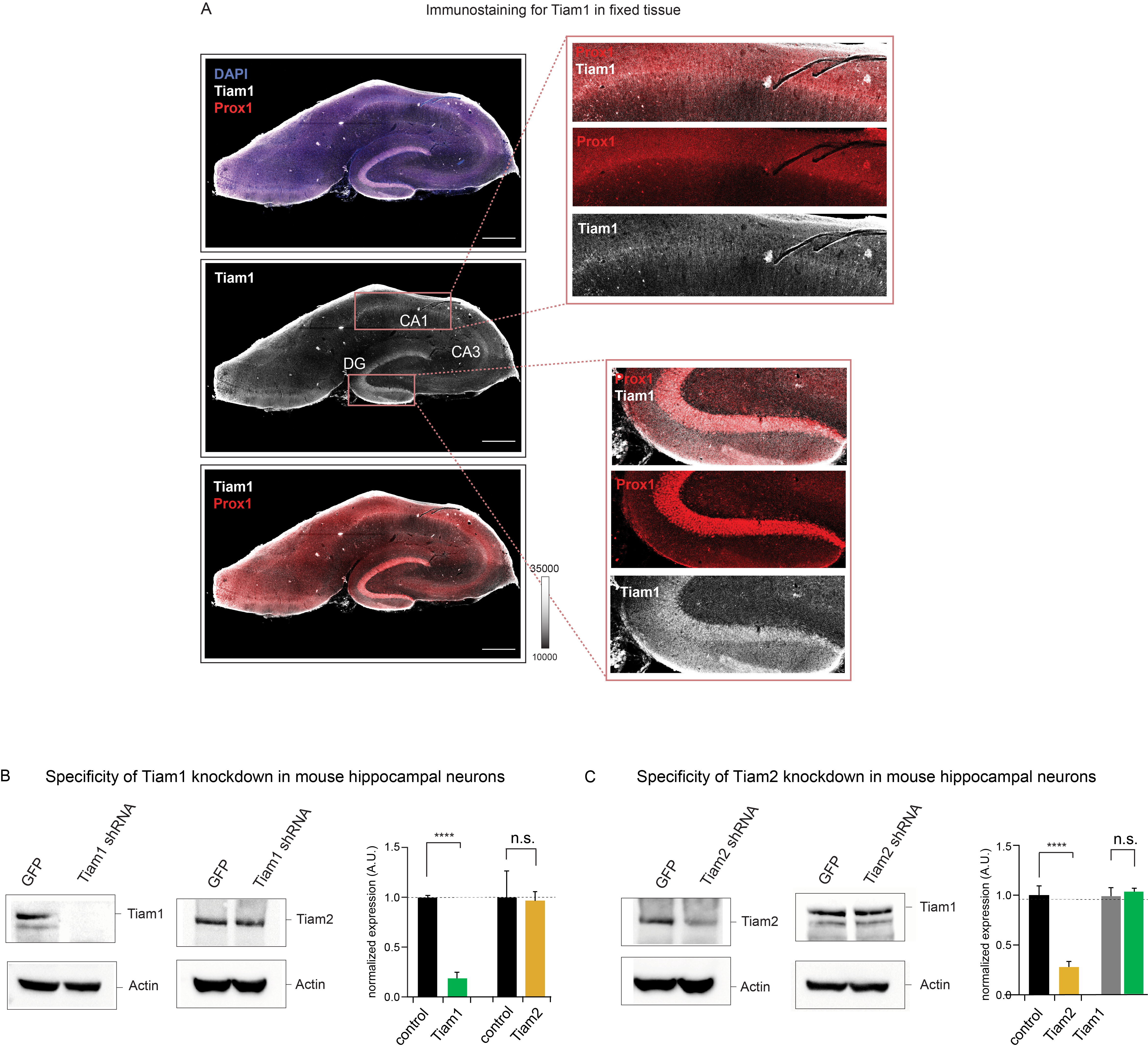

Supplement: Figure 4-1 — A Tiam1 antibody validation Tiam1 immunolabelling (white) in whole hippocampal slice co-localized with DG granule neuron specific Prox1 (red) and DAPI (blue), (inset) DG granule cell body layer (scale bar: 500μm). B Western blot showing shRNA-mediated reduction of Tiam1 and unaltered Tiam2 expression in Tiam1 shRNA expressing mouse hippocampal neurons, C Western blot showing shRNA-mediated reduction of Tiam2 and unaltered Tiam1 expression in Tiam2 shRNA expressing mouse hippocampal neurons. Barplots show mean ± SEM of experimental replicates (Tiam1 shRNA: Tiam1 n = 5, Tiam2 n = 4, Tiam2 shRNA: Tiam2 n = 4, Tiam1 n = 3, ****p < 0.0001, Mann- Whitney test). Download Figure 4-1, TIF file. [file eneuro-11-ENEURO.0500-21.2024-s004.tif]
